# An overview of the consortia, non‐profit groups and other organizations in the Alzheimer's advocacy space over the past quarter century

**DOI:** 10.1002/alz70859_100312

**Published:** 2025-12-25

**Authors:** Phyllis Barkman Ferrell

**Affiliations:** ^1^ Davos Alzheimer’s Collaborative, Wayne, PA USA

## Abstract

An Overview of the Consortia and Collaborative Initiatives, the Alzheimer's Research and Advocacy Space Over the Past Quarter Century

**Background:**

25 years ago, the number of organizations focusing on Alzheimer’s research and advocacy was few and were often operating in geographic and functional silos. The Alzheimer’s Association (founded in 1980), Alzheimer’s Disease International (1984), the Alzheimer’s Disease Research Centers (1984), the Consortium to Establish a Registry for Alzheimer’s Disease (1986), the Alzheimer’s Disease Cooperative Study (1991), and the National Alzheimer’s Coordinating Center (1999) were the most prominent U.S. organizations, with a heavy focus on research. In Europe, organizations had a focus on policy, research funding, and care advocacy. Alzheimer’s Europe (1990) drew member organizations together in Brussels to discuss pan‐EU policy needs. Alzheimer’s Research UK (1992) and Alzheimer’s Society UK (1979), though both located in the UK, had outsized impact across Europe. In Japan, a super‐aging society, the Health and Global Policy Institute, one of the largest international think tanks, has provided policy recommendations to the Japanese government since 2004.

**Methods:**

Not only have the number and types of organizations expanded substantially, the silos have also begun to break down as groups have combined together on pre‐competitive projects that span geographies and function to move the field forward faster and more collaboratively. We reviewed major consortia and summarized the projects by type and goals. Additionaly, we provide concept and network maps to show interconnectivity and influence between organizations, key stakeholders and funders.

**Results:**

Dozens of consortia have been working collaboratively to improve the lives of individuals with Alzheimer's disease and related dementias. Public, private and philanthropic entities provide funding and other support for projects with a priority for data sharing and collective action. Advocacy organizations use the output to effect policy and clinical practice change and increase awareness and support to move the field forward faster for patients and their loved ones.

**Conclusions:**

Awareness of other organizations and their goals and efforts will reduce redundant efforts and will facilitate collaboration and progress. This awareness sets the stage for accelerated benefits for those impacted by ADRD over the next 25 years.

